# Seroepidemiology of Bartonella vinsonii subsp. berkhoffii infection in California coyotes, 1994-1998.

**DOI:** 10.3201/eid0505.990514

**Published:** 1999

**Authors:** C Chang, K Yamamoto, B B Chomel, R W Kasten, D C Simpson, C R Smith, V L Kramer

**Affiliations:** University of California, Davis 95616, USA.

## Abstract

The prevalence of antibodies to Bartonella vinsonii subsp. berkhoffii in coyotes (Canis latrans) in California ranged from 51% in central to 34% in southern and 7% in northern California. Seropositive coyotes were more likely to be from coastal than inland counties (p clustered distribution of Bartonella seropositivity in coyotes suggests that B. vinsonii subsp. berkhoffii infection is vectorborne. Further investigation is warranted to evaluate which arthropods are vectors and what the mode of transmission is from wildlife to domestic dogs and possibly humans.


### Seroepidemiology of *Bartonella vinsonii* subsp. *berkhoffii* Infection in California Coyotes, 1994-1998[1]

**Chao-chin Chang,* Kazuhiro Yamamoto,* Bruno B. Chomel,* Rickie W. Kasten,* Darren C. Simpson,† Charles R. Smith,‡ and Vicki L. Kramer‡**

*University of California, Davis, California, USA; †Santa Clara County Department of Health Services, San Jose, California, USA; ‡California Department of Health Services, Sacramento, California, USA

The prevalence of antibodies to *Bartonella vinsonii* subsp. *berkhoffii* in coyotes (*Canis latrans*) in California ranged from 51% in central to 34% in southern and 7% in northern California. Seropositive coyotes were more likely to be from coastal than inland counties ($p < 0.05$). The clustered distribution of *Bartonella* seropositivity in coyotes suggests that *B. vinsonii* subsp. *berkhoffii* infection is vectorborne. Further investigation is warranted to evaluate which arthropods are vectors and what the mode of transmission is from wildlife to domestic dogs and possibly humans.

*Bartonella* species are small gram-negative bacteria, which have been identified in a wide range of mammalian species, including felines and rodents (1-3). Several *Bartonella* species are human pathogens (4). Bacillary angiomatosis, initially recognized in AIDS patients, was related in the early 1990s to a new bacterium, *Bartonella henselae* (5,6), which was later associated with cat scratch disease (4). Clinical entities have been associated with several *Bartonella* species in humans; for example, *B. henselae*, *B. quintana*, and *B. elizabethae* have been associated with human cases of endocarditis (7-11). *B. vinsonii*, isolated only from small rodents, has not yet been confirmed as a cause of human disease (12). *B. vinsonii* subsp. *berkhoffii*, was recently isolated from a dog with endocarditis (13). It was found that 3.6% of 1,920 dogs in North Carolina and Virginia were seropositive to *B. vinsonii* subsp. *berkhoffii* antigen (14). *Bartonella*-seropositive dogs were 5.6 times more likely to be flea infested than were seronegative dogs, but 14 times more likely to have a history of heavy tick exposure. For the dogs with known tick exposure, a high correlation was found between seroreactivity to *B. vinsonii* subsp. *berkhoffii* and seroreactivity to *Ehrlichia canis* and *Babesia canis*, both well-known tickborne infections. Furthermore, Breitschwerdt et al. (15) reported that dogs infected with *Ehrlichia* species were frequently coinfected with *B. vinsonii* subsp. *berkhoffii*, suggesting that this infection in dogs could also be tickborne.

In July 1996, a 3 1/2-year-old boy was bitten by a coyote (*Canis latrans*) in Santa Clara County, CA, and became ill with fever and lymphadenopathy. The incident prompted an investigation of possible *Bartonella* infection in the boy and coyotes. Because of the clinical signs in the child and the absence of *Francisella tularensis* antibodies in two coyotes, the coyotes were tested for *Bartonella* antibodies by an immunofluorescence test with a *B. henselae* antigen, and both tested positive (titers of 1:128 and 1:512). *B. vinsonii* subsp. *berkhoffii*, identified by both polymerase chain reaction/restriction fragment length polymorphism (PCR/RFLP) of the citrate synthase and 16S rRNA genes and by partial sequencing of the 16S rRNA gene, was isolated from several coyotes from Santa Clara County (3). The PCR/RFLP profiles of the coyote isolates and the domestic dog isolate were the same (ATCC 51672 strain).

Address for correspondence: Bruno B. Chomel, Department of Population Health and Reproduction, School of Veterinary Medicine, University of California, Davis, CA, 95616, USA; fax: 530-752-2377; e-mail: bbchomel@ucdavis.edu.

[1]An earlier version of this paper was presented at the Second International Conference on Emerging Zoonoses, Strasbourg, France, November 5–9, 1998.

To determine the geographic distribution of *B. vinsonii* subsp. *berkhoffii* infection in California coyotes, we analyzed data for age, sex, and origin of the animals to establish possible risk factors associated with seropositivity and to identify high-risk coyote populations. Since coyotes (*C. latrans*) belong to the same genus as domestic dogs (*C. familiaris*), wild canids may serve as a reservoir of *B. vinsonii* subsp. *berkhoffii*, and transmission to domestic dogs could occur through common ectoparasites.

### The Study

Coyote blood samples (nobuto filter strips or serum) collected from 34 of the 58 counties in California from 1994 to 1998 were tested serologically for *B. vinsonii* subsp. *berkhoffii* antibodies. Age, sex, county of sample collection, and collection date were recorded.

For 869 specimens, specific antibodies against a *B. vinsonii* subsp. *berkhoffii* purified antigen (outer membrane proteins) were detected by enzyme-linked immunosorbent assay (16). Cut-off values were established as described (17,18). The cut-off value (Optical Density [OD] > 0.2) for seropositivity was determined by the average OD plus three standard deviations (SD) of 76 nobuto strips from counties where all OD values were < 0.190. Statistically, animals with OD values > 0.2 can be considered to be seropositive with 99% confidence.

Univariate analysis by chi-square and Fisher exact tests was applied first to screen for factors associated with seropositivity to *B. vinsonii* subsp. *berkhoffii*. Potential confounders were evaluated by 10% change of estimates, i.e., odds ratio. Multiple logistic regression analysis was applied to evaluate the adjusted effects of the associated factors.

The overall prevalence of *B. vinsonii* subsp. *berkhoffii*-seropositive coyotes was 35% (95% confidence interval [CI]: 25% to 48%). *Bartonella* antibody prevalence varied from 51% in central California to 34% in southern and 7% in northern California (Table 1). The prevalence of seropositive coyotes from coastal counties was significantly higher than that of seropositive coyotes from inland counties (Figure) in all three geographic areas ($p < 0.05$). No gender difference in seropositivity was detected. Antibody prevalence was higher in adult coyotes (37%) than in young coyotes (< 1 year of age) (29%) (Table 2). The prevalence of high antibody titers (OD > 0.5) was

Table 1. Seroprevalence of *Bartonella vinsonii* subsp. *berkhoffii* infection in 869 coyotes, California, 1994–1998

| Geographic region | County | No. seropositive | (%) |
|---|---|---|---|
| Northern California | | 13/174 | (7) |
| Coastal counties | Humboldt | 2/12 | (17) |
| | Mendocino | 7/30 | (23) |
| Inland counties | Siskiyou | 0/26 | (0) |
| | Modoc | 0/47 | (0) |
| | Lassen | 1/7 | (14) |
| | Plumas | 1/40 | (3) |
| | Butte | 0/2 | (0) |
| | Trinity | 0/1 | (0) |
| | Shasta | 2/9 | (22) |
| Central California | | 180/355 | (51) |
| Coastal counties | Solano | 0/4 | (0) |
| | Sonoma | 1/3 | (33) |
| | Alameda | 14/32 | (44) |
| | Santa Clara | 116/167 | (69) |
| | Santa Cruz | 1/1 | (100) |
| Inland counties | Sierra | 6/32 | (19) |
| | Nevada | 2/10 | (20) |
| | Placer | 0/1 | (0) |
| | El Dorado | 26/63 | (41) |
| | Alpine | 0/2 | (0) |
| | Calaveras | 0/2 | (0) |
| | Tuolumne | 4/8 | (50) |
| | Mono | 0/7 | (0) |
| | Mariposa | 4/7 | (57) |
| | Merced | 2/2 | (100) |
| | Sacramento | 3/3 | (100) |
| | Madera | 1/11 | (9) |
| Southern California | | 115/340 | (34) |
| Coastal counties | Monterey | 4/10 | (40) |
| | San Luis Obispo | 18/56 | (32) |
| | Santa Barbara | 27/45 | (60) |
| | Ventura | 0/1 | (0) |
| | Los Angeles | 15/26 | (58) |
| | San Diego | 6/6 | (100) |
| Inland counties | Kern | 41/190 | (22) |
| | San Bernardino | 4/6 | (67) |

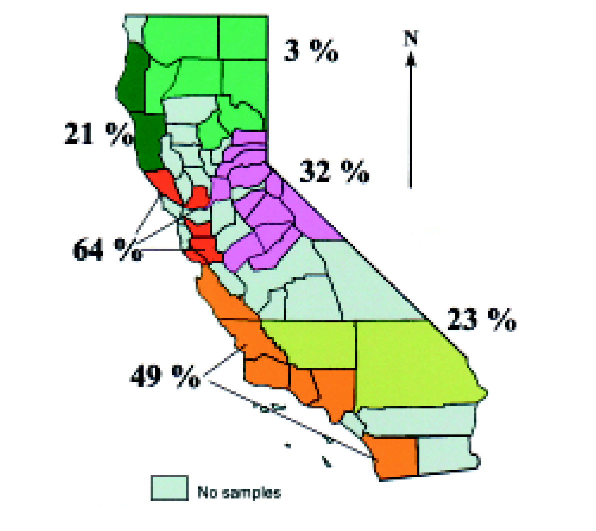

Figure. *Bartonella vinsonii* subsp. *berkhoffii* seroprevalence in 869 California coyotes (1994–1998).

Table 2. Seroprevalence of *Bartonella vinsonii* subsp. *berkhoffii* infection, by age, sex, and season, for 869 coyotes, California, 1994–1998

| Characteristic | No. seropositive (%) |
|---|---|
| Age[a] | |
| <1 year old | 35/122 (29) |
| ≥1 year old | 273/745 (37) |
| Sex[b] | |
| Female | 155/407 (38) |
| Male | 152/455 (33) |
| Season[c] | |
| Spring (Mar to May) | 86/296 (29) |
| Summer (Jun to Aug) | 121/286 (42) |
| Fall (Sep to Nov) | 67/182 (37) |
| Winter (Dec to Feb) | 27/85 (32) |

[a]Not available for two coyotes.
[b]Not available for seven coyotes.
[c]Not available for 20 coyotes.

Table 3. Annual *Bartonella vinsonii* subsp. *berkhoffii* antibody prevalence in 869 coyotes, by age group, California, 1994–1998

| Year | All ages no. (%) | ≥1 year old no. (%) | <1 year old no. (%) |
|---|---|---|---|
| 1994 | 22/62 (35) | 21/61 (34) | 1/1 (100) |
| 1995 | 55/159 (35) | 53/146 (36) | 2/13 (15) |
| 1996[a] | 47/124 (37) | 37/100 (37) | 9/23 (39) |
| 1997[a] | 115/275 (42) | 107/238 (45) | 8/36 (19) |
| 1998 | 70/249 (28) | 55/200 (28) | 15/49 (31) |

[a]Age not available for one coyote.

similar in adults (20 [7.3%] of 273) and young coyotes (3 [8.6 %] of 35). There were no major differences in prevalence by age groups for each of the three following OD groups (OD > 0.2 and ≤ 0.3 [young coyotes, 37%; adults, 48%]; OD > 0.3 and ≤ 0.4 [young coyotes, 40%; adults, 27%]; and OD > 0.4 and ≤ 0.5 [young coyotes, 14%; adults; 19%]). Summer (June to August) had the highest prevalence (42%) of *Bartonella*-seropositive coyotes and spring had the lowest (29%) (Table 2). However, in young coyotes, antibody prevalence increased from 23% (3/13) in winter and 24% (6/25) in spring to 28% (12/43) in summer and 33% (13/39) in fall. Of the 25 coyotes < 6 months old, neither of the two captured in spring were seropositive, whereas 20% of the 10 captured in summer and 46% of the 13 captured in fall were seropositive.

Antibody prevalence was relatively constant during the 5-year period, from a low of 28% in 1998 to a high of 42% in 1997 (Table 3). In 1997, the prevalence was significantly higher in adults than in young coyotes (odds ratio [OR] = 2.86, 95% CI = 1.2, 7.5). This difference was associated with the high prevalence of positive adults from coastal central (50 [82%] of 61) and southern (20 [77%] of 26) California. That year, none of the young coyotes from inland areas were seropositive, but 5 (62%) of 8 and 4 (80%) of 5 from coastal central and southern California were positive. Overall, infection occurred more frequently in young coyotes from coastal central and southern California (29 [57%] of 51) than from inland areas (5 [15%] of 34) (OR = 7.65, 95% CI = 2.3, 26.9).

By multiple logistic regression analysis, after adjustment for age, odds ratios for central California and southern California were 9.6 (95% CI: 5.2, 17.7) and 5.2 (95% CI: 2.8, 9.7) times higher, respectively, than for northern California. The adjusted odds ratio for the coastal counties compared with inland counties was 3.7 (95% CI: 2.7, 5.0). Similarly, coyotes ≥ 1 year old were 60% more likely to be seropositive (OR = 1.6, 95% CI = 1.0, 2.6) than young coyotes.

### Conclusions

Because the discovery of *B. vinsonii* subsp. *berkhoffii* is so recent, little is known about the epidemiology and the mode of transmission of this organism in domestic dogs and wild canids. Of 54 coyotes from Santa Clara County, 16 (30%) were *B. vinsonii* subsp. *berkhoffii* bacteremic (19). Therefore, coyotes could serve as a potential reservoir for *B. vinsonii* subsp. *berkhoffii*, which could also be transmitted to domestic dogs, either by mechanical means (biting and scratching) or through arthropod vectors. *Bartonella* spp. are usually transmitted by arthropod vectors. Cats are the main reservoir for *B. henselae* and cat fleas are the main vector for transmission between cats (20,21). No direct transmission of *B. henselae* from cat to cat has been documented experimentally (22,23). Therefore, *B. vinsonii* subsp. *berkhoffii* transmission among coyotes and between coyotes and dogs is more likely to be vectorborne. Furthermore, the seasonal trends of *Bartonella* antibody prevalence in coyotes, especially young coyotes, indicate a moderate seasonal peak in summer and fall compared with winter and spring, when ectoparasites are the most abundant (14).

The high *Bartonella* antibody prevalence, especially in young coyotes, in coastal areas of central and southern California, indicates active transmission in these counties compared with the low prevalence of infection in young coyotes in inland areas. The clustered distribution of infection in young coyotes in California may be associated with the mode of transmission of the infection, possibly through arthropods.

Limited data on the actual distribution of various arthropods throughout California are available. As suggested by Pappalardo et al. (14) and Breitschwerdt et al. (15), ticks could be potential vectors for *Bartonella* transmission in domestic dogs. The clustered distribution of *Bartonella* infection in California coyotes shown in this study seems to coincide with the known geographic distribution of tick species (*Ixodes pacificus* and *Dermacentor variabilis*) that can feed on carnivores in California (24). According to the studies of Chaniotis et al. (25) and Ayala et al. (26), the distribution of sandflies in California is mainly in the Upper Sonoran zone of central California. This distribution could also match the coastal range of *Bartonella* infection in coyotes. Since sandflies are well-known vectors for *B. bacilliformis* transmission, they could also be considered for *Bartonella* spp. transmission in coyotes. Nevertheless, further studies need to be conducted to verify this hypothesis. In domestic carnivores, flea infestation is common and widespread in most of California. Cat fleas (*Ctenocephalides felis*) have been shown to be responsible for *B. henselae* infection in cats, and they also can infest domestic dogs and possibly coyotes. Although flea transmission cannot be completely ruled out, the dog study conducted in the Eastern United States showed that ten control dogs with heavy flea infestation but no known tick exposure had no seropositivity to *B. vinsonii* subsp. *berkhoffii* (14).

Only a few infectious agents can be transmitted by multiple vectors, e.g., tularemia and relapsing fever. Therefore, further investigation will be necessary to determine which of all the potential vectors mentioned above can harbor *B. vinsonii* subsp. *berkhoffii* under natural conditions and which one plays a major role for *B. vinsonii* subsp. *berkhoffii* transmission in canids. Furthermore, it is necessary to evaluate the risk of domestic dog infection with *Bartonella* spp. from wild canids and the potential risk of transmission to humans.

### Acknowledgments

The authors thank the California County Health Offices and their trappers for providing blood samples from coyotes as part of the California Plague Surveillance Program and Pamela K. Swift for initiating the coyote testing.

Dr. Chang is a doctoral student in Epidemiology at the University of California, Davis, under the direction of Dr. Bruno B. Chomel. His research interests include molecular epidemiology of *Bartonella* infections and potential vectors for *Bartonella* spp. transmission.

### References

1. Birtles RJ, Fichet-Calvet E, Raoult D, Ashford RW. Detection and genotypic differentiation of *Bartonella* species infecting a Tunisian *Psammomys obesus* population. Proceedings from 13th Sesqui-Annual Meeting of the American Society for Rickettsiology; 1997 Sep 21-24; Champion, Pennsylvania. Abstract 34.
2. Heller R, Kubina M, Delacour G, Mahoudeau I, Lamarque F, Artois M, et al. Prevalence of *Bartonella* spp. in blood of wild small rodents. Abstracts of the 97th General Meeting of the American Society for Microbiology; 1997 May 4-7;Miami Beach, Florida. Washington: The Society, 1997. p. 115.
3. Chomel BB, Kasten RW, Chang CC, Yamamoto K, Heller R, Maruyama S, et al. Isolation of *Bartonella* spp. from California wildlife. Abstracts of the International Conference on Emerging Infectious Diseases; 1998 Mar 8-11; Atlanta, Georgia. p. 21.10.
4. Anderson BE, Neuman MA. *Bartonella* spp. as emerging human pathogens. Clin Microbiol Rev 1997;10:203-19.
5. Slater LN, Welch DF, Min KW. *Rochalimaea henselae* causes bacillary angiomatosis and peliosis hepatitis. Arch Intern Med 1992;152:602-6.
6. Welch DF, Pickett DA, Slater LN, Steigerwalt AG, Brenner DJ. *Rochalimaea henselae* sp. nov., a cause of septicemia, bacillary angiomatosis, and parenchymal bacillary peliosis. J Clin Microbiol 1992;30:275-80.
7. Daly JS, Worthington MG, Brenner DJ, Moss CW, Hollis DG, Weyant RS, et al. *Rochalimaea elizabethae* sp. nov. isolated from a patient with endocarditis. J Clin Microbiol 1993;31:872-81.
8. Spach DH, Callis KP, Paauw DS, Houze YB, Schoenknecht FD, Welch DF, et al. Endocarditis caused by *Rochalimaea quintana* in a patient infected with human immunodeficiency virus. J Clin Microbiol 1993;31:692-4.
9. Holmes AH, Greenough TC, Balady GJ, Regnery RL, Anderson BE, O'Keane JC, et al. *Bartonella henselae* endocarditis in an immunocompetent adult. Clin Infect Dis 1995;21:1004-7.
10. Drancourt M, Mainardi JL, Brouqui P, Vandenesch F, Carta A, Lehnert F, et al. *Bartonella* (*Rochalimaea*) *quintana* endocarditis in three homeless men. N Engl J Med 1995;332:419-23.
11. Drancourt M, Birtles R, Chaumentin G, Vandenesch F, Etienne J, Raoult D. New serotype of *Bartonella henselae* in endocarditis and cat-scratch disease. Lancet 1996;347:441-3.
12. Baker JA. A rickettsial infection in Canadian voles. J Exp Med 1946;84:37-50.
13. Breitschwerdt EB, Kordick DL, Malarkey DE, Keene B, Hadfield TL, Wilson K. Endocarditis in a dog due to infection with a novel *Bartonella* subspecies. J Clin Microbiol 1995;33:154-60.

14. Pappalardo BL, Correa MT, York CC, Peat CY, Breitschwerdt EB. Epidemiologic evaluation of the risk factors associated with exposure and seroreactivity to *Bartonella vinsonii* in dogs. Am J Vet Res 1997;58:467-71.
15. Breitschwerdt EB, Hegarty BC, Hancock SI. Sequential evaluation of dogs naturally infected with *Ehrlichia canis*, *Ehrlichia chaffeensis*, *Ehrlichia equi*, *Ehrlichia ewingii*, or *Bartonella vinsonii*. J Clin Microbiol 1998;36:2645-51.
16. Chomel BB, Carlos ET, Kasten RW, Yamamoto K, Chang CC, Carlos RS, et al. *Bartonella henselae* and *Bartonella clarridgeiae* infection in domestic cats in the Philippines. Am J Trop Med Hyg 1999;60:593-7.
17. Richardson MD, Turner A, Warnock DW, Llewellyn PA. Computer assisted rapid enzyme linked immunosorbent assay (ELISA) in the serological diagnosis of Aspergillosis. J Immunol Methods 1983;56:201-7.
18. Magnarelli LA, Anderson CS, Apperson CS, Fish D, Johnson RC, Chappell A. Spirochetes in ticks and antibodies to *Borrelia burgdorferi* in white-tailed deer from Connecticut, New York State, and North Carolina. J Wildl Dis 1986;22:178-88.
19. Chang CC, Yamamoto K, Chomel BB, Kasten RW, Simpson D, Wilson B. Sero-epidemiology of *Bartonella* infection in wild canids in California. Proceedings of the International Conference on Emerging Zoonoses; 1998 Nov 5-9; Strasbourg, France. p. 58.
20. Koehler JE, Glaser CA, Tappero JW. *Rochalimaea henselae* infection—a new zoonosis with the domestic cat as reservoir. JAMA 1994;271:531-5.
21. Chomel BB, Kasten RW, Floyd-Hawkins K, Chi B, Yamamoto K, Roberts-Wilson J, et al. Experimental transmission of *Bartonella henselae* by the cat flea. J Clin Microbiol 1996;34:1952-6.
22. Abbott RC, Chomel BB, Kasten RW, Floyd-Hawkins KA, Kikuchi Y, Koehler JE, et al. Experimental and natural infection with *Bartonella henselae* in domestic cats. Comp Immunol Microbiol Infect Dis 1997;20:41-51.
23. Guptill L, Slater L, Wu CC, Lin TL, Glickman LT, Welch DF, et al. Experimental infection of young specific pathogen-free cats with *Bartonella henselae*. J Infect Dis 1997;176:206-16.
24. Furman DP, Loomis EC. The ticks of California (Acari: Ixodida). Berkeley: University of California press, 1984.
25. Chaniotis BN, Anderson JR. Age structure, population dynamics and vector potential of *Phlebotomus* in northern California. II. Field population dynamics and natural flagellate infections in parous females. J Med Entomol 1968;5:273-92.
26. Ayala SC, Lee D. Saurian malaria: development of sporozoites in two species of phlebotomine sandflies. Science 1970;167:891-2.
